# LncRNA SOX21‐AS1 Promotes the Progression of Pancreatic Cancer by Sponging miR‐9‐3p and Upregulating YOD1

**DOI:** 10.1002/kjm2.70054

**Published:** 2025-06-17

**Authors:** Han‐Bing Xu, Jian‐Tao Han, Cheng‐Peng Zhang, Bin Jiang

**Affiliations:** ^1^ Department of Hepatobiliary Pancreatic Gastrointestinal Surgery Wuhan Third Hospital Wuhan China; ^2^ Department of Gastrointestinal Abdominal Wall and Hernia Surgery Wuhan Third Hospital Wuhan China

**Keywords:** miR‐9‐3p, pancreatic cancer, SOX21‐AS1, YOD1

## Abstract

Pancreatic cancer (PC) is a highly aggressive malignancy of the digestive system. Recent studies have indicated that the long noncoding RNA SOX21‐AS1 is significantly upregulated in PC tissue samples. This study aims to elucidate the biological role and underlying molecular mechanisms of SOX21‐AS1 in PC progression. Quantitative real‐time PCR (qRT‐PCR) and western blot analyses were employed to assess RNA and protein expression levels, respectively. The subcellular localization of SOX21‐AS1 was determined using subcellular fractionation assays. PC cell viability, migratory capacity, and apoptosis were evaluated through CCK‐8 assays, wound healing assays, and flow cytometry. Dual‐luciferase reporter and RNA pull‐down assays were conducted to confirm the interactions between miR‐9‐3p and either SOX21‐AS1 or YOD1. Additionally, a xenograft mouse model was established to investigate the in vivo effects of SOX21‐AS1. The findings revealed that SOX21‐AS1 is highly expressed in PC tissues and cell lines, with its upregulation correlating with poor patient prognosis. Functional assays demonstrated that knockdown of SOX21‐AS1 suppressed PC cell proliferation and migration, induced apoptosis in vitro, and reduced tumor growth in vivo. Mechanistically, SOX21‐AS1 competitively interacted with miR‐9‐3p to upregulate YOD1, consequently activating the TGF‐β/Smad signaling pathway. Furthermore, overexpression of YOD1 reversed the tumor‐suppressive effects observed after SOX21‐AS1 knockdown. In conclusion, SOX21‐AS1 promotes PC cell malignancy through the miR‐9‐3p/YOD1 axis and subsequent activation of TGF‐β/Smad signaling.

## 1 Introduction

Pancreatic cancer (PC) is a highly lethal malignancy of the digestive system and is the third leading cause of cancer‐related mortality worldwide [1]. Numerous risk factors contribute to the pathogenesis of PC, including advanced age, genetic mutations, chronic pancreatitis, and exposure to trace elements [2]. Although substantial progress has been made in the diagnosis and treatment of PC, the overall efficacy of current therapies remains limited, primarily due to the disease's nonspecific early symptoms and aggressive metastatic behavior [3]. Approximately 90% of PC cases are diagnosed at a late stage, after they have spread beyond the pancreas, leading to poor clinical outcomes [4]. Currently used chemotherapeutic agents, such as gemcitabine, 5‐fluorouracil, oxaliplatin, and nab‐paclitaxel, offer limited benefits, primarily due to the emergence of drug resistance [5]. In recent years, newer therapeutic approaches, including immunotherapy and targeted therapies, have been explored. However, their clinical efficacy has fallen short of expectations [6]. Consequently, there is an urgent need to identify reliable biomarkers and elucidate the molecular mechanisms underlying PC to facilitate the development of more effective therapeutic strategies.

Long noncoding RNAs (lncRNAs) represent a class of RNA transcripts that lack protein‐coding potential [7]. They have gained increasing attention as key regulators of gene expression, playing essential roles in numerous biological and pathological processes, including cell proliferation, apoptosis, and migration [8]. Emerging evidence has suggested that lncRNAs can function either as oncogenes or tumor suppressors, depending on the cancer context [9]. In PC, many lncRNAs are aberrantly upregulated compared to normal pancreatic tissues, contributing to disease progression. For instance, lncRNA UCA1 is markedly elevated in PC tissues and has been identified as an independent prognostic indicator for poor clinical outcomes [10]. Similarly, Wang et al. demonstrated that LINC00941 overexpression promotes PC cell proliferation and metastasis [11]. Additionally, FAM83A‐AS1 has been implicated in enhancing the malignant phenotype of PC cells and has potential utility as a diagnostic biomarker [12]. Collectively, these findings underscore the critical role of lncRNAs in the development and progression of PC.

The lncRNA SOX21 antisense RNA1 (SOX21‐AS1), located on chromosome 13q32.1, has been implicated in the pathogenesis of various diseases, including several types of cancer. For instance, SOX21‐AS1 has been shown to facilitate osteosarcoma cell proliferation and invasion by modulating the miR‐7‐5p/IRS2 signaling axis [13]. Additionally, silencing SOX21‐AS1 has been reported to attenuate the aggressive behavior of glioma cells [14]. Notably, increased expression of SOX21‐AS1 has also been observed in PC samples [15]. Despite these findings, the specific molecular mechanisms through which SOX21‐AS1 affects the malignant phenotype of PC cells remain to be fully elucidated.

Emerging evidence indicates that the regulatory functions of lncRNAs are closely associated with their subcellular localization. In the cytoplasm, lncRNAs often function as competing endogenous RNAs (ceRNAs), whereby they upregulate target messenger RNAs (mRNAs) by competitively binding to shared microRNAs (miRNAs), which bind to the 3′ untranslated regions (3′UTRs) of mRNAs [16]. This study aimed to investigate the functional role and molecular mechanisms of SOX21‐AS1 in PC. It was hypothesized that SOX21‐AS1 may act as an oncogenic lncRNA in PC by promoting cell proliferation and migration while inhibiting apoptosis through a ceRNA‐mediated regulatory network. The significance of these findings lies in identifying SOX21‐AS1 as a potential oncogenic driver in PC, contributing to disease progression through miRNA sponging and downstream signaling activation. This mechanistic insight not only enhances our understanding of lncRNA‐mediated regulation in PC but also offers a promising therapeutic target for future intervention strategies.

## 2 Methods and Materials

### 2.1 Clinical Specimens

A total of 60 paired PC and adjacent non‐tumorous tissue samples were obtained from patients who underwent surgical resection at the Hospital between April 2021 and April 2024. All patients included in the study had not received any radiotherapy or chemotherapy prior to surgery. Individuals with significant dysfunction of vital organs (heart, liver, or kidneys), a history of autoimmune disorders, or ongoing acute or chronic infectious diseases were excluded from the study. Written informed consent was obtained from each participant prior to sample collection. The tissue specimens were promptly frozen in liquid nitrogen and stored at −80°C. The study protocol was reviewed and approved by the Ethics Committee of the Hospital.

### 2.2 Cell Culture

The human PC cell lines (BxPC‐3, SW1990, and PANC‐1) were purchased from American Type Culture Collection (ATCC, Manassas, VA, USA). BxPC‐3 cells were cultured in RPMI‐1640 medium (ATCC, 30‐2001), while SW1990 and PANC‐1 cells were maintained in Dulbecco's Modified Eagle Medium (DMEM; ATCC, 30‐2002). The immortalized normal human pancreatic ductal epithelial cell line (HPDE) was procured from CMBIO (Shanghai, China) and cultured in DMEM. All culture media were supplemented with 10% fetal bovine serum (FBS; ATCC, 30‐2020). Cells were incubated in a humidified atmosphere at 37°C with 5% CO_2_.

### 2.3 Cell Transfection

To downregulate SOX21‐AS1 expression, specific short hairpin RNAs (shRNAs) targeting SOX21‐AS1 (sh‐SOX21‐AS1#1 and sh‐SOX21‐AS1#2) and a negative control shRNA (sh‐NC) were used. For YOD1 overexpression, the full‐length coding sequence of YOD1 was cloned into the pcDNA3.1 vector (pcDNA3.1/YOD1), with the empty pcDNA3.1 vector serving as the control. To upregulate miR‐9‐3p, synthetic miR‐9‐3p mimics and corresponding scrambled negative control mimics (NC mimics) were employed. All plasmids and oligonucleotides were obtained from GenePharma (Shanghai, China). Transfections were carried out in BxPC‐3 or PANC‐1 cells using Lipofectamine 2000 (Invitrogen, Carlsbad, CA, USA) with the following final concentrations: 40 nM for sh‐SOX21‐AS1#1/2 and sh‐NC, 50 nM for miR‐9‐3p mimics and NC mimics, and 20 nM for pcDNA3.1/YOD1 and pcDNA3.1 vectors. Cells were harvested 48 h post‐transfection for subsequent analyses.

### 2.4 Quantitative Real‐Time Polymerase Chain Reaction (qRT‐PCR) Analysis

Total RNA was isolated from cultured cells and tumor tissue samples using TRIzol reagent (B0201, HaiGene, Harbin, China), according to the manufacturer's instructions. Complementary DNA (cDNA) synthesis was performed using the PrimeScript RT Reagent Kit (RR047A, Takara, Beijing, China) for SOX21‐AS1 and mRNAs, and the Mir‐X miRNA First‐Strand Synthesis Kit (638,313, Takara) for miRNAs. Quantitative real‐time PCR (qRT‐PCR) was carried out using SYBR Green Master Mix (Q131‐02/03, Vazyme, Nanjing, China) on the 7500 Real‐Time PCR System (4,351,151, Thermo Fisher Scientific, Waltham, MA, USA). The qPCR thermal cycling conditions were as follows: an initial denaturation at 95°C for 10 min, followed by 40 cycles of denaturation at 95°C for 15 s and extension at 72°C for 15 s. GAPDH was used as the internal control for SOX21‐AS1 and YOD1, while U6 served as the reference for miR‐9‐3p. Relative gene expression levels were calculated using the 2^−ΔΔCT^ method [17]. The primer sequences are listed below:

SOX21‐AS1.

Forward (F): 5′‐GCTGCAGGAGAGTTAAGGA‐3′.

Reverse (R): 5′‐CTCTCCACTCGCCTAAACC‐3′.

miR‐9‐3p.

F: 5′‐TCTTTGGTTATCTAGCTGTAT‐3′.

R: 5′‐GAACATGTCTGCGTATCTC‐3′.

YOD1.

F: 5′‐CATCCAATCTGGTGACATGC‐3′.

R: 5′‐GCACCACGTTTAGTAAATGCA‐3′.

U6.

F: 5′‐ATACAGAGAAAGTTAGCACGG‐3′.

R: 5′‐GGAATGCTTCAAAGAGTTGTG‐3′.

GAPDH.

F: 5′‐AACGTGTCAGTGGTGGACCTG‐3′.

R: 5′‐AGTGGGTGTCGCTGTTGAAGT‐3′.

### 2.5 Subcellular Fractionation Assay

The subcellular localization of SOX21‐AS1 was assessed using a subcellular fractionation assay, following the manufacturer's protocol. Total RNA was isolated from the nuclear and cytoplasmic compartments using the Cytoplasmic & Nuclear RNA Purification Kit (Norgen Biotek, Canada). The relative expression levels of SOX21‐AS1 in each fraction were quantified by qRT‐PCR. U6 and GAPDH were used as internal controls for the nuclear and cytoplasmic fractions, respectively.

### 2.6 Cell Counting Kit‐8 (CCK‐8) Assay

BxPC‐3 or PANC‐1 cells were seeded into 96‐well plates at a density of 3 × 10^3^ cells per well. After incubation for 24, 48, or 72 h, Cell Counting Kit‐8 (CCK‐8; A311‐01/02, Vazyme) solution was added to each well, followed by an additional 4‐h incubation period. Cell viability was assessed by measuring the absorbance at 450 nm using a microplate reader (Thermo Fisher Scientific). The resulting absorbance values were used to generate cell viability curves.

### 2.7 Wound Healing Assay

The transfected PC cells were seeded into six‐well plates at a density of 5 × 10^5^ cells per well. Once the cell monolayer reached approximately 70% confluence, a straight‐line scratch was made across the surface using a sterile pipette tip to create a wound. The wells were then rinsed three times with PBS to remove detached cells. An inverted microscope (Olympus, Tokyo, Japan) was used to capture images of the wound area, and cell migration was evaluated by measuring the distance between the wound edges at 0 and 24 h.

### 2.8 Flow Cytometry Analysis

The transfected cells (4 × 10^6^ cells/ml) were harvested by centrifugation for 5 min, after which the culture medium was discarded. The cell pellets were washed with PBS and subsequently fixed in 70% ethanol for 1 h. Following fixation, the cells were resuspended in 3 mL of PBS and incubated for 5 min. Annexin V and propidium iodide (PI; Invitrogen) were then added to the cell suspension, and staining was performed in the dark for 10 min. Apoptotic cells were subsequently analyzed using a FACS Calibur flow cytometer (BD Biosciences, Shanghai, China).

### 2.9 Western Blot Analysis

Proteins were extracted from cultured cells and tumor tissues using radioimmunoprecipitation assay (RIPA) buffer (PH0316, PHYGENE, Fuzhou, China). Protein concentrations were determined using the bicinchoninic acid (BCA) assay. Equal amounts of protein were separated by SDS‐PAGE and subsequently transferred onto polyvinylidene fluoride (PVDF) membranes. The membranes were then blocked with 5% nonfat milk and incubated overnight at 4°C with gentle agitation using the following primary antibodies: cleaved caspase 3 (ab2302, 1/500), Bax (ab32503, 1/5000), Bcl‐2 (ab32124, 1/1000), YOD1 (DF4597, 1 mg/mL), TGF‐β (ab179695, 1/1000), p‐Smad2/3 (ab272332, 1/1000), Smad2/3 (ab202445, 1/1000), α‐SMA (ab124964, 1/10000), fibronectin (ab268020, 1/1000), collagen I (ab255809, 1/1000), and GAPDH (ab181602, 1/10000) (all from Abcam (Shanghai, China) except anti‐YOD1 (Affinity Biosciences, Liyang, China)). After washing with PBST (YT8034, YITA, China), membranes were incubated with the appropriate secondary antibody (ab98624, 1:5000, Abcam). Protein bands were visualized using an enhanced chemiluminescence (ECL) detection kit (Thermo Fisher Scientific) and quantified with ImageJ software.

### 2.10 Dual‐Luciferase Reporter Assay

The wild‐type (Wt) fragments of SOX21‐AS1 and the YOD1 3′UTR containing the predicted miR‐9‐3p binding sites were amplified and inserted into the pmirGLO vector (Promega, Madison, WI, USA) to construct SOX21‐AS1‐Wt and YOD1 3′UTR‐Wt reporter plasmids. Mutant constructs (SOX21‐AS1‐Mut and YOD1 3′UTR‐Mut) were generated by introducing point mutations into the miR‐9‐3p binding sites using the QuickMutation Site‐Directed Mutagenesis Kit (Beyotime, Shanghai, China). These reporter plasmids were co‐transfected into BxPC‐3 or PANC‐1 cells along with either miR‐9‐3p mimics or NC mimics using Lipofectamine 2000. After 48 h, luciferase activity was measured using the Dual‐Luciferase Reporter Assay System (Promega, USA). Firefly luciferase activity was normalized to Renilla luciferase activity to calculate relative luciferase expression levels.

### 2.11 RNA Pull‐Down Assay

The RNA pull‐down assay was performed using the Pierce Magnetic RNA‐Protein Pull‐Down Kit (Thermo Fisher Scientific), following the manufacturer's instructions. In brief, biotin‐labeled miR‐9‐3p probe (Bio‐miR‐9‐3p) and a negative control probe (Bio‐NC) were incubated with streptavidin‐coated magnetic beads in 500 μL of binding buffer for 1 h. The probe‐conjugated beads were then incubated with cell lysates at 4°C overnight under constant rotation. After incubation, the RNA‐protein complexes were eluted, and the associated RNA was extracted for subsequent qRT‐PCR analysis.

### 2.12 In Vivo Experiments

Ten male BALB/c nude mice (5 weeks old) were obtained from Cavens (Changzhou, China) and maintained in a specific pathogen‐free (SPF) facility under controlled environmental conditions (temperature: 21°C ± 2°C; humidity: 50%–60%) with a 12‐h light/dark cycle. Following a 1‐week acclimatization period, the mice were randomly assigned into two groups: sh‐NC and sh‐SOX21‐AS1#1 (*n* = 5 per group). Each mouse was subcutaneously injected in the right flank with 1 × 10^6^ PANC‐1 cells previously transfected with either sh‐NC or sh‐SOX21‐AS1#1. Tumor growth was monitored weekly, and tumor volume was calculated using the formula: 0.5 × length × width^2^. After 4 weeks, all mice were euthanized by cervical dislocation under anesthesia, and tumor tissues were harvested for further analysis. All animal experiments were conducted in accordance with protocols approved by the Ethics Committee of Wuhan Third Hospital.

### 2.13 Statistical Analysis

GraphPad Prism 8 software was used to perform statistical analysis. The data are expressed as the mean ± standard deviation (SD) from at least three independent experiments. Differences between two groups were assessed using the unpaired two‐tailed Student's *t*‐test, while one‐way ANOVA followed by Tukey's post hoc test was applied for multiple group comparisons. Pearson correlation analysis was performed to examine the relationships among SOX21‐AS1, miR‐9‐3p, and YOD1 expression levels in PC samples. A *p* value less than 0.05 was considered statistically significant.

## 3 Results

### 3.1 SOX21‐AS1 Is Upregulated in PC and Is Associated With Poor Prognosis

**FIGURE 1:**
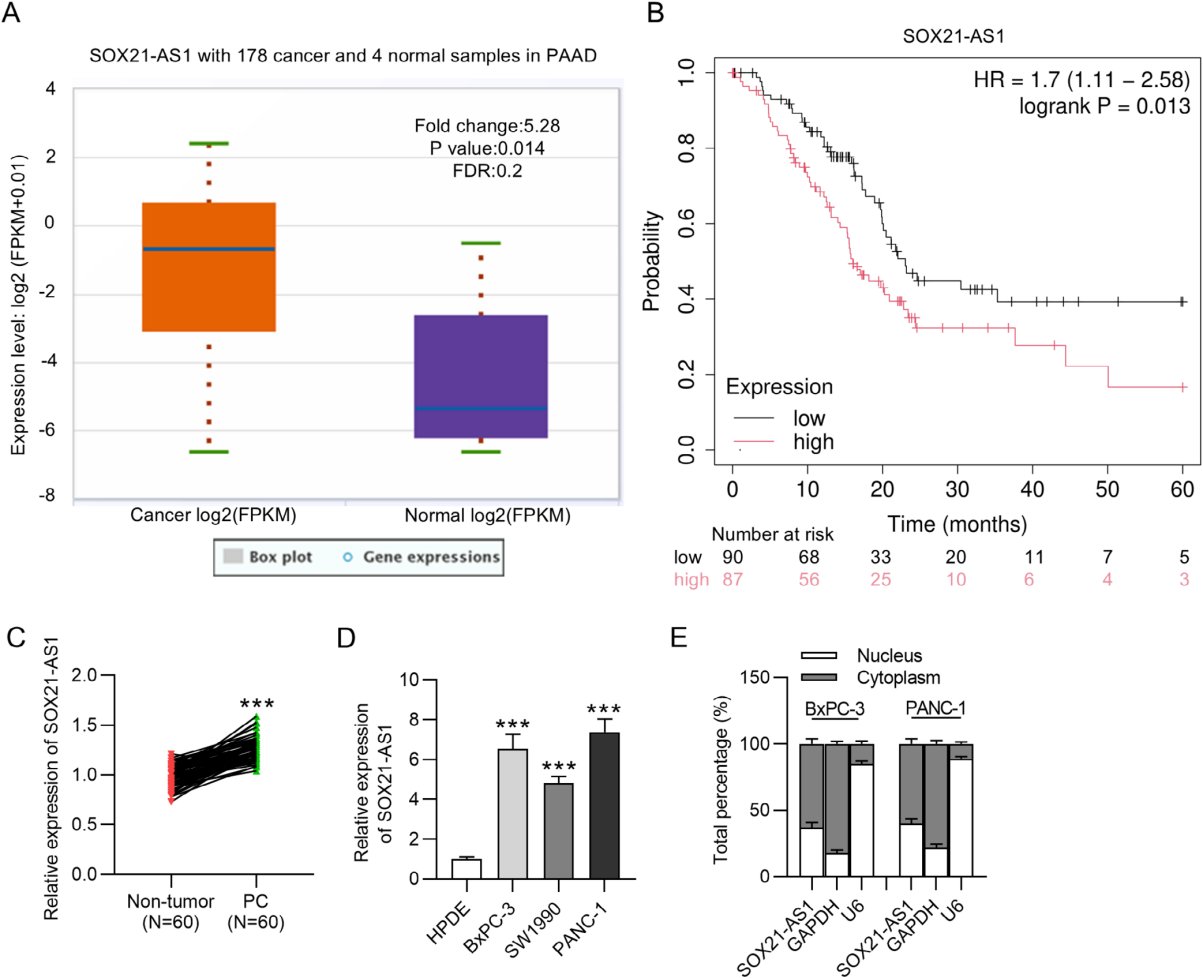
Upregulation of SOX21‐AS1 in PC and its association with poor prognosis of PC patients. (A) Analysis of SOX21‐AS1 expression in PC tumor tissues versus normal tissues using the ENCORI database (https://starbase.sysu.edu.cn/). (B) Kaplan–Meier survival analysis showing the association between SOX21‐AS1 expression levels and overall survival in PC patients. (C) qRT‐PCR analysis of SOX21‐AS1 expression in paired PC and adjacent non‐tumorous tissue samples (*N* = 60 pairs). (D) Relative expression of SOX21‐AS1 in PC cell lines and the non‐tumor HPDE cell line measured by qRT‐PCR. (E) Subcellular localization of SOX21‐AS1 in BxPC‐3 and PANC‐1 cells determined by subcellular fractionation assay. ***p < 0.001.

The data retrieved from the ENCRIO database (https://starbase.sysu.edu.cn/) revealed that SOX21‐AS1 is significantly upregulated in PC tumor tissues (*n* = 178) compared to normal tissues (*n* = 4) (Figure 1A). Kaplan–Meier survival analysis further demonstrated that PC patients with elevated SOX21‐AS1 expression exhibited a poorer prognosis than those with lower expression levels (Figure 1B). Consistently, our experimental results confirmed higher SOX21‐AS1 expression in PC tissue samples relative to adjacent non‐tumorous tissues (Figure 1C). qRT‐PCR analysis also showed that SOX21‐AS1 expression was markedly elevated in PC cell lines compared to the normal HPDE cells, with the highest expression observed in BxPC‐3 and PANC‐1 cells (Figure 1D). Consequently, these two cell lines were selected for subsequent experiments. Additionally, subcellular fractionation analysis revealed that SOX21‐AS1 is predominantly localized in the cytoplasm of BxPC‐3 and PANC‐1 cells (Figure 1E), suggesting a potential post‐transcriptional regulatory role. Collectively, these findings indicate that SOX21‐AS1 is upregulated in PC cells and tissues and that its elevated expression is associated with poor clinical outcomes in PC patients.

### 3.2 SOX21‐AS1 Depletion Inhibits the Viability and Migration but Promotes the Apoptosis of PC Cells

**FIGURE 2:**
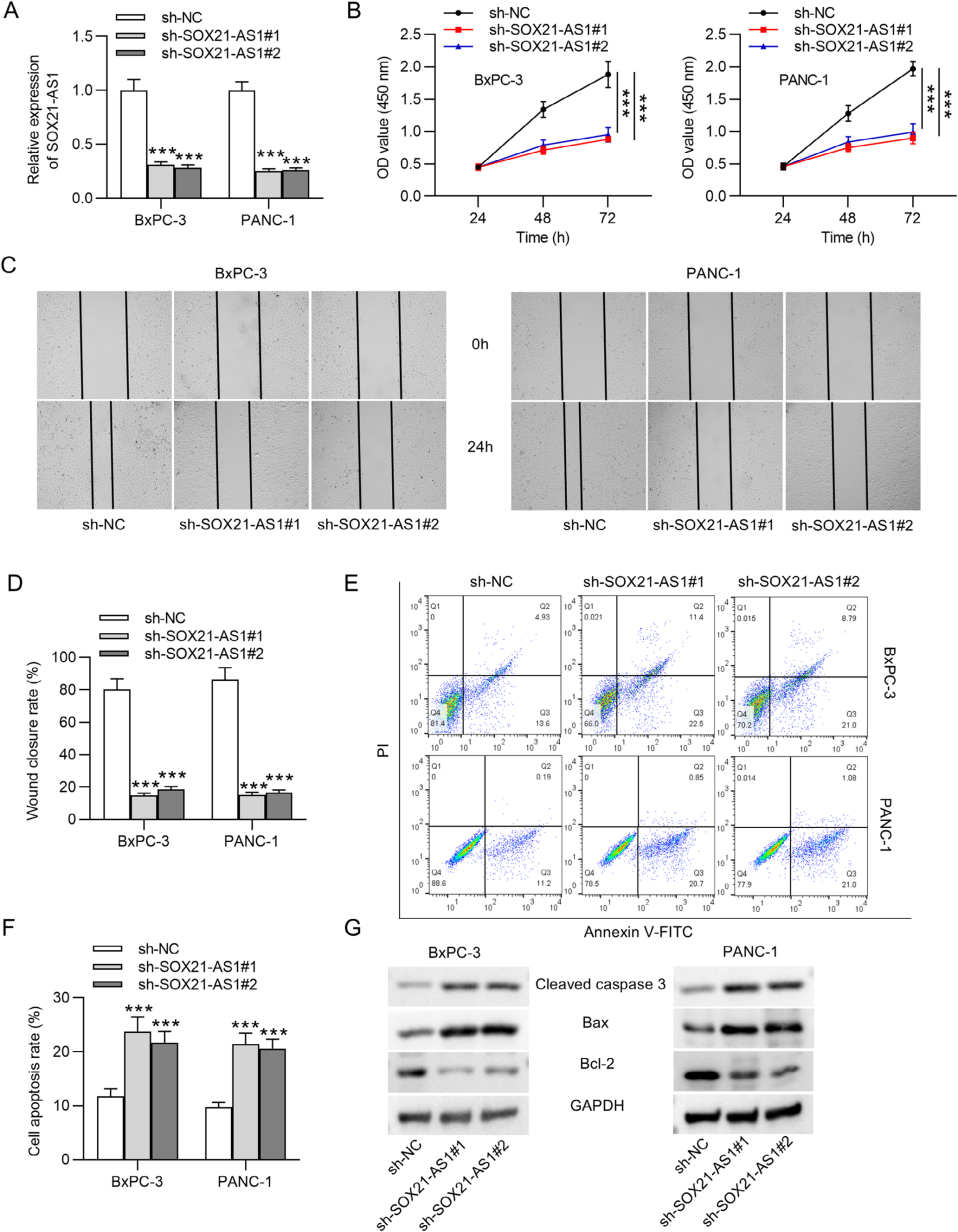
SOX21‐AS1 depletion suppressed the malignant phenotypes of PC cells. Loss‐of‐function experiments were performed in BxPC‐3 and PANC‐1 cells transfected with sh‐NC or sh‐SOX21‐AS1#1/2. (A) qRT‐PCR confirmed approximately 70% knockdown efficiency of SOX21‐AS1. (B) CCK‐8 assay showed reduced cell viability following SOX21‐AS1 silencing. (C and D) Wound healing assay demonstrated impaired migratory ability in SOX21‐AS1‐depleted cells. (E and F) Flow cytometry analysis revealed increased apoptosis upon SOX21‐AS1 knockdown. (G) Western blot analysis indicated elevated levels of cleaved caspase‐3 and Bax and reduced Bcl‐2 expression in cells with SOX21‐AS1 knockdown. ***p < 0.001.

Based on the findings above, the biological function of SOX21‐AS1 in PC cells was further investigated. BxPC‐3 and PANC‐1 cells were transfected with sh‐SOX21‐AS1#1 or sh‐SOX21‐AS1#2 to achieve knockdown of SOX21‐AS1. As shown in Figure 2A, SOX21‐AS1 expression was significantly reduced in both sh‐SOX21‐AS1 groups compared to the sh‐NC group, with approximately 70% knockdown efficiency. To explore the functional consequences of SOX21‐AS1 silencing, a series of loss‐of‐function assays were performed. CCK‐8 assays revealed that SOX21‐AS1 knockdown significantly suppressed the viability of BxPC‐3 and PANC‐1 cells (Figure 2B). In the wound healing assay, silencing of SOX21‐AS1 markedly impaired the migratory capacity of these cells (Figure 2C,D). Apoptosis levels were evaluated by flow cytometry, which demonstrated that depletion of SOX21‐AS1 led to a significant increase in apoptotic cell populations (Figure 2E,F). Furthermore, western blot analysis showed that SOX21‐AS1 knockdown elevated the expression of pro‐apoptotic proteins cleaved caspase‐3 and Bax, while reducing the expression of the anti‐apoptotic protein Bcl‐2 (Figure 2G). Taken together, these results suggest that SOX21‐AS1 can promote the malignant behavior of PC cells by enhancing cell viability and migration while inhibiting apoptosis. Its downregulation can impair these oncogenic properties, highlighting its potential as a therapeutic target in PC.

### 3.3 SOX21‐AS1 Binds to miR‐9‐3p in PC Cells

**FIGURE 3:**
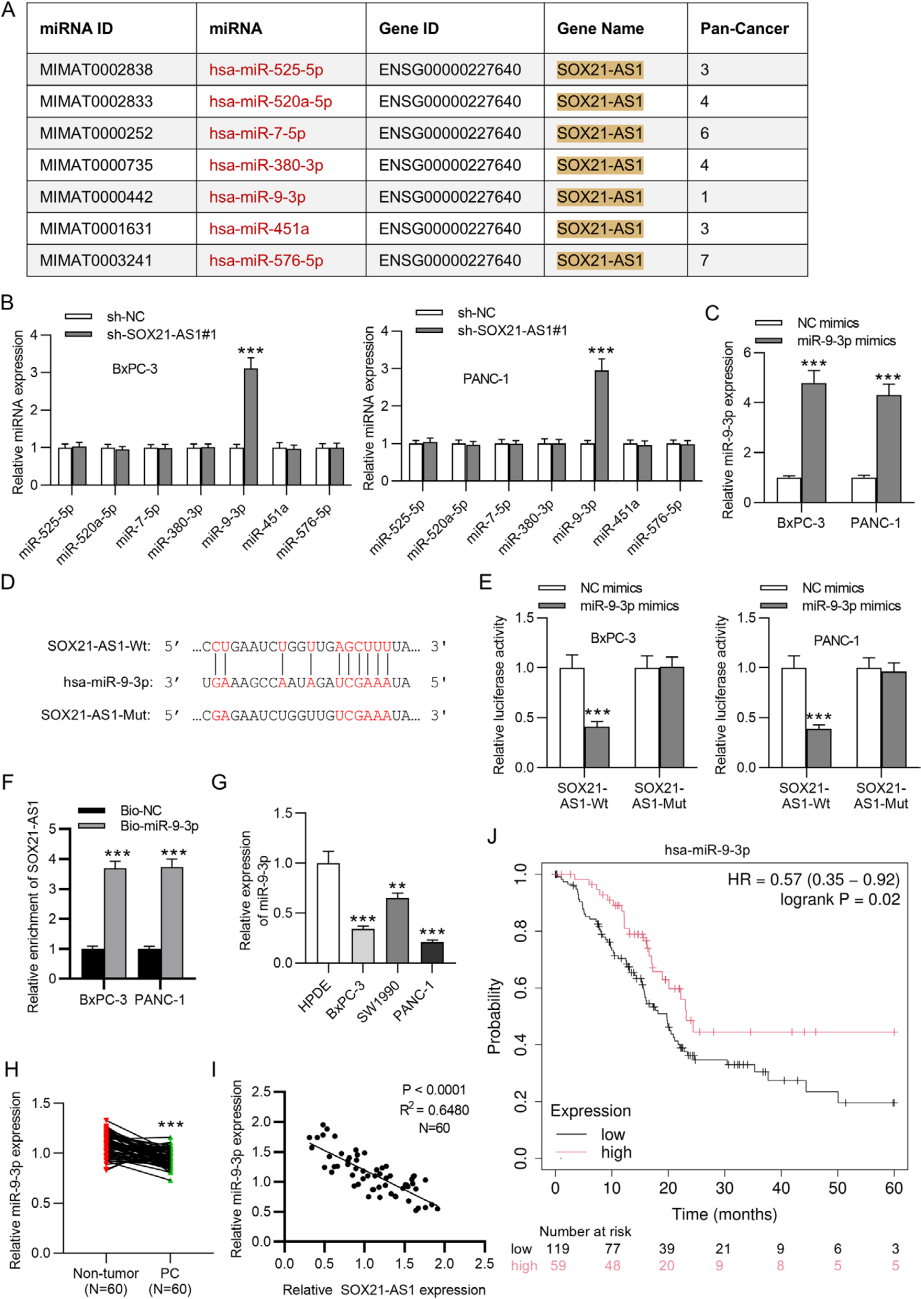
SOX21‐AS1 functions as a molecular sponge for miR‐9‐3p in PC cells. (A) Prediction of potential downstream miRNAs of SOX21‐AS1 using the ENCORI database. (B) qRT‐PCR analysis of miRNA expression levels (miR‐525‐5p, miR‐520a‐5p, miR‐7‐5p, miR‐380‐3p, miR‐9‐3p, miR‐451a, and miR‐576‐5p) in cells transfected with sh‐NC or sh‐SOX21‐AS1#1. (C) Verification of miR‐9‐3p overexpression efficiency by qRT‐PCR. (D) Predicted binding site between SOX21‐AS1 and miR‐9‐3p identified via ENCORI. (E) Dual‐luciferase reporter assay confirmed the specific interaction between SOX21‐AS1 and miR‐9‐3p. (F) RNA pull‐down assay validated the direct binding of SOX21‐AS1 to miR‐9‐3p. (G) qRT‐PCR analysis of miR‐9‐3p expression in PC and non‐tumor cell lines. (H) Expression levels of miR‐9‐3p in 60 paired PC and adjacent normal tissue samples. (I) Pearson correlation analysis revealed a negative correlation between miR‐9‐3p and SOX21‐AS1 expression in PC tissues (*N* = 30). (J) Kaplan–Meier survival analysis showing that low miR‐9‐3p expression is associated with poorer prognosis in PC patients. **p < 0.01, ***p < 0.001.

Given the previous observation that SOX21‐AS1 was predominantly localized in the cytoplasm of PC cells, it was hypothesized that SOX21‐AS1 may exert its regulatory function by interacting with specific miRNAs. To investigate this, the ENCORI database was utilized to identify potential downstream miRNAs of SOX21‐AS1. Using the filtering criteria of CLIP ≥ 1 and pan‐cancer ≥ 1, seven candidate miRNAs were identified (Figure 3A). Among these, miR‐9‐3p was the only miRNA found to be significantly upregulated in PC cells following transfection with sh‐SOX21‐AS1#1 (Figure 3B), suggesting a potential inverse regulatory relationship. Consequently, miR‐9‐3p was selected for further investigation. To confirm its overexpression, PC cells were transfected with miR‐9‐3p mimics. qRT‐PCR analysis showed that miR‐9‐3p expression increased 4‐fold compared to the control group (Figure 3C). To confirm the interaction between miR‐9‐3p and SOX21‐AS1, the predicted miR‐9‐3p binding site within SOX21‐AS1 was mutated (Figure 3D), and a dual‐luciferase reporter assay was conducted. The results demonstrated that miR‐9‐3p mimics significantly reduced the luciferase activity of the wild‐type SOX21‐AS1 (SOX21‐AS1‐Wt), whereas no significant change was observed in the mutant construct (SOX21‐AS1‐Mut), indicating specific binding of miR‐9‐3p to SOX21‐AS1 (Figure 3E). To validate transfection efficiency, qRT‐PCR was performed to measure SOX21‐AS1 and miR‐9‐3p expression in co‐transfected cells. SOX21‐AS1 levels remained consistent across all co‐transfection groups (Figure S1A), while miR‐9‐3p was markedly overexpressed in cells transfected with miR‐9‐3p mimics, with no significant differences observed between the two mimic groups or between the negative controls (Figure S1B). Further supporting this interaction, RNA pull‐down assays showed a significant enrichment of SOX21‐AS1 in the Bio‐miR‐9‐3p group compared to the Bio‐NC group, confirming direct binding between SOX21‐AS1 and miR‐9‐3p (Figure 3F). Additionally, qRT‐PCR analysis revealed that miR‐9‐3p expression was significantly downregulated in PC tissues and cell lines compared to their corresponding controls (Figure 3G,H). Pearson correlation analysis identified a significant negative correlation between SOX21‐AS1 and miR‐9‐3p expression in PC tissue samples (Figure 3I). Kaplan–Meier survival analysis further indicated that low miR‐9‐3p expression was associated with poor prognosis in PC patients (Figure 3J). Collectively, these results demonstrate that SOX21‐AS1 can directly bind to and negatively regulate miR‐9‐3p in PC.

### 3.4 YOD1 Can Serve as a Downstream Target of miR‐9‐3p in PC Cells

**FIGURE 4:**
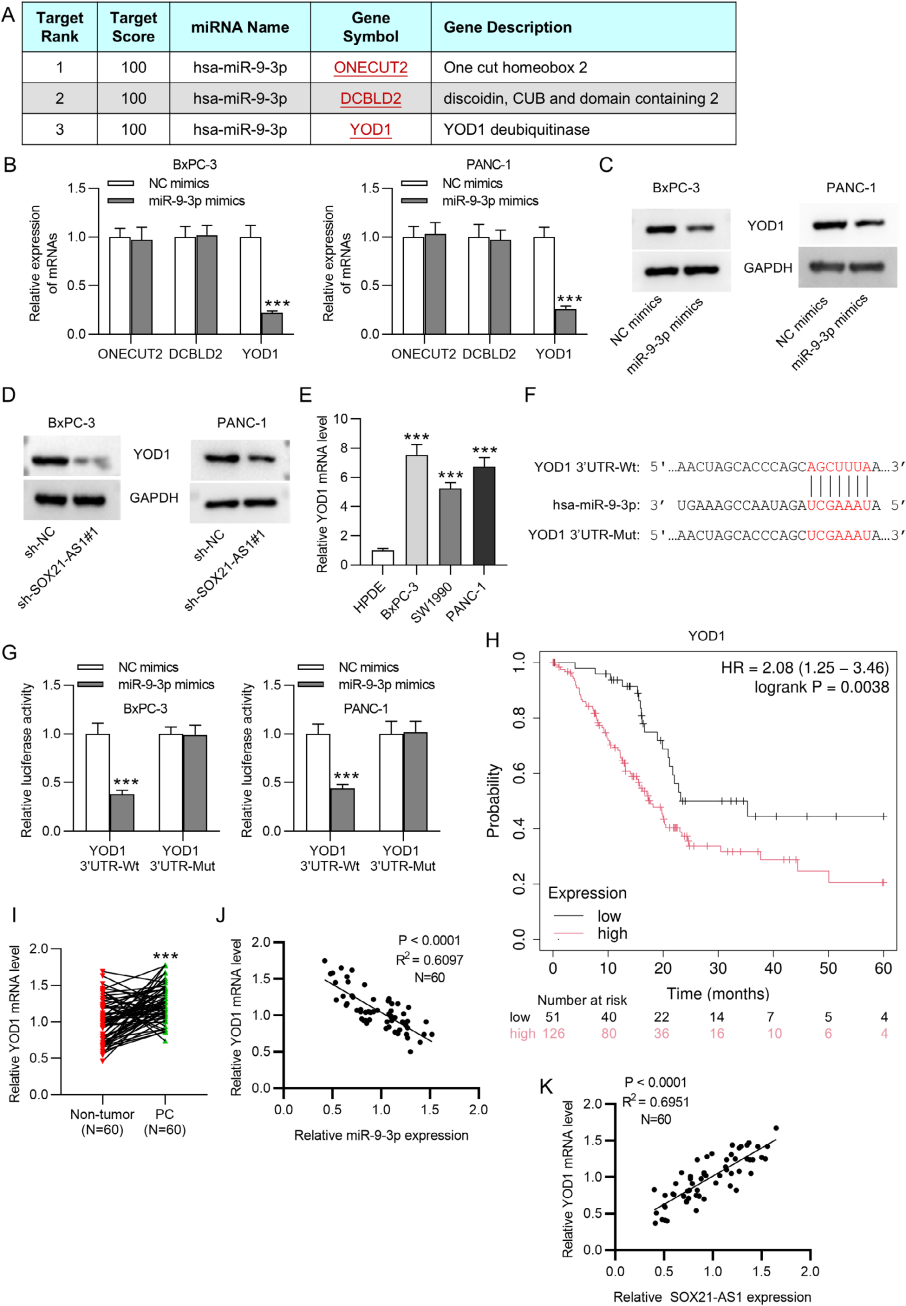
YOD1 was identified as a direct target of miR‐9‐3p. (A) Prediction of potential downstream mRNAs of miR‐9‐3p using the miRDB database. (B) qRT‐PCR analysis of ONECUT2, DCBLD2, and YOD1 mRNA levels in PC cells transfected with either NC mimics or miR‐9‐3p mimics. (C and D) Western blot analysis showing reduced YOD1 protein expression following miR‐9‐3p overexpression (C) or SOX21‐AS1 knockdown (D). (E) qRT‐PCR analysis of YOD1 expression in PC cell lines compared to non‐tumor HPDE cells. (F) Predicted binding site between miR‐9‐3p and the YOD1 3′UTR. (G) Dual‐luciferase reporter assay confirmed the direct interaction between miR‐9‐3p and YOD1. (H) Kaplan–Meier survival analysis revealed that high YOD1 expression is associated with poor prognosis in PC patients. (I) YOD1 mRNA expression levels in 60 paired PC and adjacent normal tissue samples. (J and K) Pearson correlation analysis showed a negative correlation between miR‐9‐3p and YOD1 expression (J), and a positive correlation between SOX21‐AS1 and YOD1 expression (K) in PC tissues. ***p < 0.001.

To further validate the ceRNA hypothesis, miRDB was employed to predict potential downstream target mRNAs of miR‐9‐3p. Among the top candidates identified were ONECUT2, DCBLD2, and YOD1 (Figure 4A). Subsequent qRT‐PCR analysis revealed that transfection with miR‐9‐3p mimics significantly reduced the mRNA levels of YOD1, while having no notable effect on ONECUT2 or DCBLD2 expression (Figure 4B). Based on these results, YOD1 was selected for further investigation. Western blot analysis showed that YOD1 protein levels were markedly decreased following either miR‐9‐3p overexpression or SOX21‐AS1 silencing (Figure 4C,D). Additionally, YOD1 was found to be more highly expressed in PC cell lines compared to the non‐tumor HPDE cell line (Figure 4E). The predicted miR‐9‐3p binding site within the YOD1 3′UTR is illustrated in Figure 4F. Dual‐luciferase reporter assays confirmed this interaction: miR‐9‐3p mimics significantly suppressed the luciferase activity of the YOD1 3′UTR‐Wt, but not the mutant construct (YOD1 3′UTR‐Mut) (Figure 4G). Consistent with these findings, YOD1 expression was elevated in PC tissues compared to adjacent non‐tumor tissues (Figure 4H). Furthermore, Pearson correlation analysis demonstrated that YOD1 expression was inversely correlated with miR‐9‐3p levels and positively correlated with SOX21‐AS1 levels in PC tissues (Figure 4I,J), supporting the notion that SOX21‐AS1 upregulates YOD1 by competitively binding to miR‐9‐3p. Kaplan–Meier survival analysis further revealed that high YOD1 expression was associated with poor prognosis in PC patients (Figure 4K).

### 3.5 YOD1 Overexpression Reverses SOX21‐AS1 Silencing‐Mediated Effects on the Malignant Phenotypes of PC Cells

**FIGURE 5:**
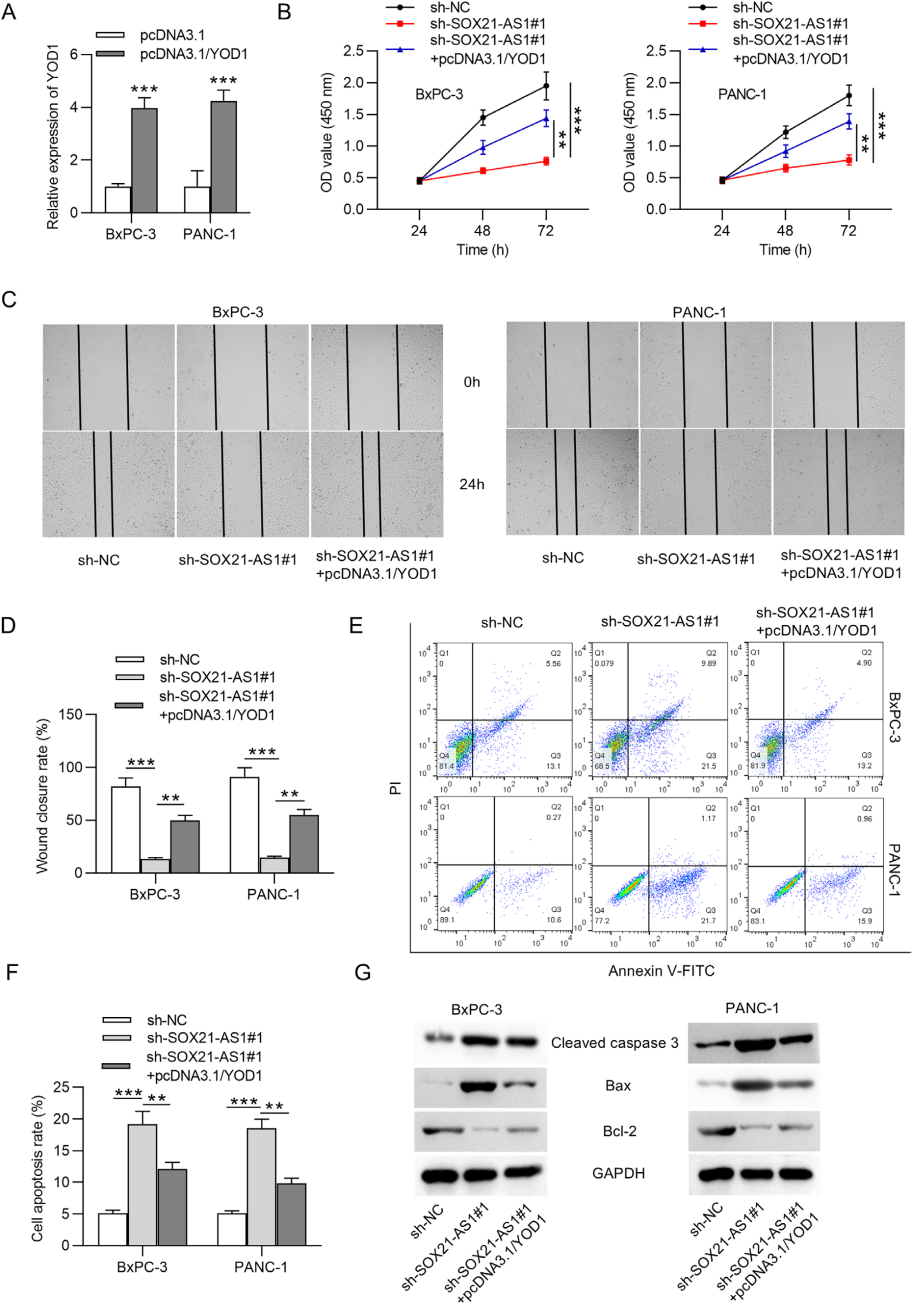
YOD1 overexpression reversed SOX21‐AS1 depletion‐mediated effects on the malignant phenotypes of PC cells. (A) qRT‐PCR analysis confirmed efficient overexpression of YOD1 after transfection with pcDNA3.1/YOD1. (B) CCK‐8 assay showed that YOD1 overexpression rescued the reduced viability of PC cells caused by SOX21‐AS1 knockdown. (C and D) Wound healing assay demonstrated that the impaired migratory ability induced by SOX21‐AS1 silencing was restored by YOD1 upregulation. (E and F) Flow cytometry analysis revealed that YOD1 overexpression attenuated the increase in apoptosis resulting from SOX21‐AS1 depletion. (G) Western blot analysis showed that the SOX21‐AS1 knockdown‐induced increase in cleaved caspase‐3 and Bax, along with the decrease in Bcl‐2, was reversed by YOD1 overexpression. **p < 0.01, ***p < 0.001.

Rescue experiments were performed to determine whether SOX21‐AS1 mediates its oncogenic effects through the miR‐9‐3p/YOD1 axis in PC cells. To this end, YOD1 was overexpressed by transfecting cells with the pcDNA3.1/YOD1 plasmid, resulting in a more than four‐fold increase in YOD1 expression (Figure 5A). CCK‐8 assays revealed that YOD1 overexpression effectively reversed the reduction in cell viability caused by SOX21‐AS1 knockdown (Figure 5B). Similarly, the suppression of cell migratory capacity induced by SOX21‐AS1 silencing was restored upon YOD1 upregulation (Figure 5C,D). Flow cytometry analysis demonstrated that YOD1 overexpression also attenuated the pro‐apoptotic effect of SOX21‐AS1 knockdown (Figure 5E,F). Furthermore, western blot analysis showed that the increased expression of cleaved caspase‐3 and Bax, along with decreased Bcl‐2 expression induced by SOX21‐AS1 silencing, was reversed upon YOD1 upregulation (Figure 5G). Taken together, these results confirm that SOX21‐AS1 can promote the malignant phenotype of PC cells at least in part through upregulation of YOD1, thereby highlighting the functional significance of the SOX21‐AS1/miR‐9‐3p/YOD1 regulatory axis in PC progression.

### 3.6 SOX21‐AS1 Activates the TGF‐β/Smad2/3 Signaling Pathway via Upregulating YOD1

**FIGURE 6:**
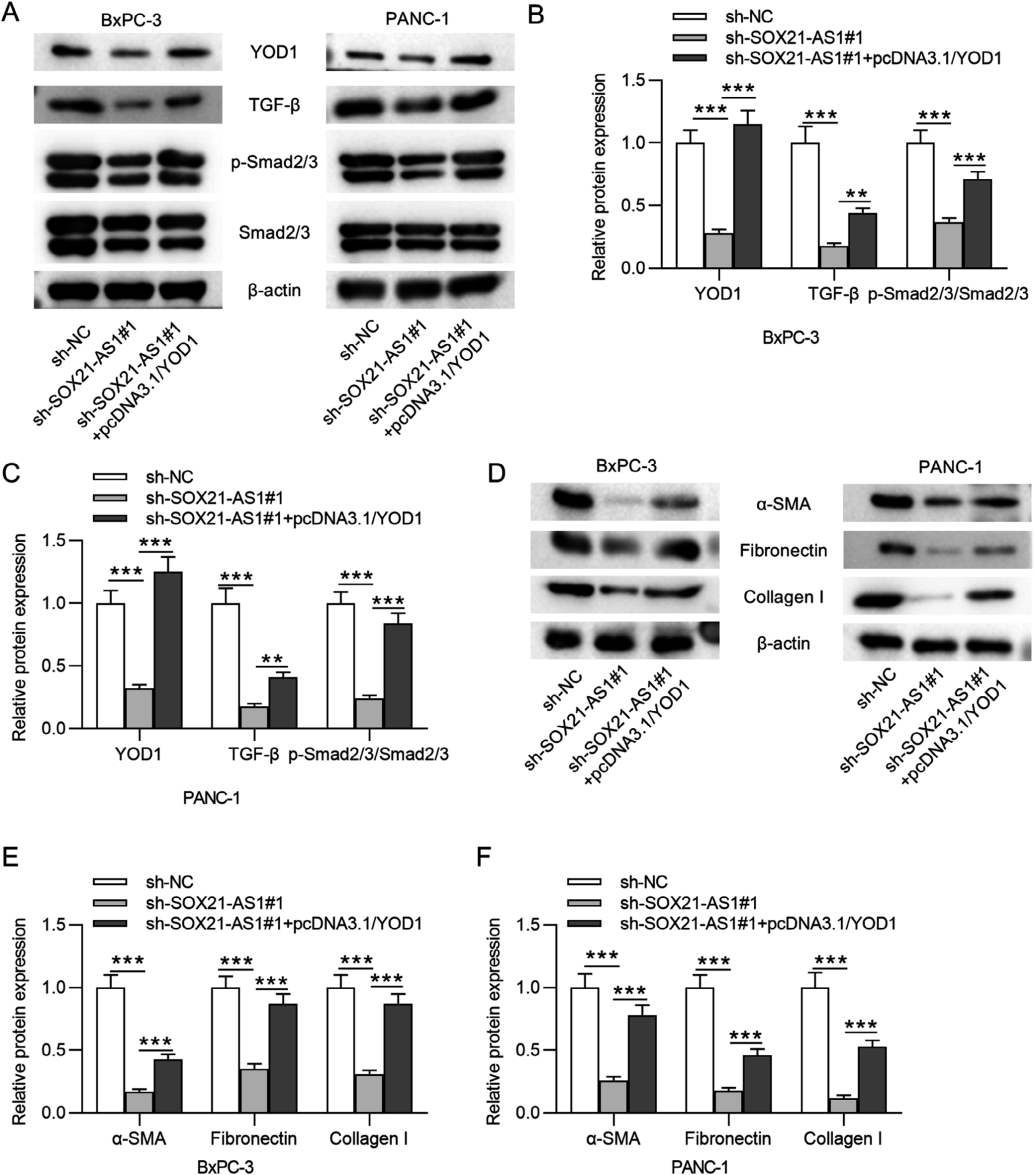
SOX21‐AS1 activated the TGF‐β/Smad2/3 signaling pathway by upregulating YOD1. (A–C) Western blot analysis of YOD1, TGF‐β, and p‐Smad2/3 protein levels in PC cells after transfection with the indicated constructs. (D–F) Western blot analysis of desmoplasia‐related markers, including α‐SMA, fibronectin, and collagen I, in response to SOX21‐AS1 knockdown and YOD1 overexpression. **p < 0.01, ***p < 0.001.

To further elucidate the mechanism by which SOX21‐AS1 contributes to the aggressiveness of PC cells, we investigated its effect on the TGF‐β/Smad2/3 signaling pathway, which has been reported to be regulated by YOD1. Western blot analysis revealed that silencing SOX21‐AS1 led to a marked reduction in the protein levels of YOD1, TGF‐β, and phosphorylated Smad2/3 (p‐Smad2/3) (Figure 6A–C). Conversely, overexpression of YOD1 reversed these inhibitory effects, restoring the expression of these proteins. Consistent with the protein data, qRT‐PCR analysis also showed similar trends in YOD1 and TGF‐β mRNA levels (Figure S2). To assess the downstream impact of this pathway, we examined the expression of fibrosis‐related markers, namely α‐SMA, fibronectin, and collagen I, which are key mediators of desmoplasia, a hallmark feature of PC. As shown in Figure 6D–F, SOX21‐AS1 knockdown suppressed the expression of these proteins, while YOD1 overexpression effectively reversed this suppression. Overall, these findings suggest that SOX21‐AS1 can enhance the malignant behavior of PC cells by promoting YOD1 expression and activating the TGF‐β/Smad2/3 signaling cascade, thereby contributing to desmoplastic progression in PC.

### 3.7 SOX21‐AS1 Knockdown Inhibits Tumor Growth In Vivo

**FIGURE 7:**
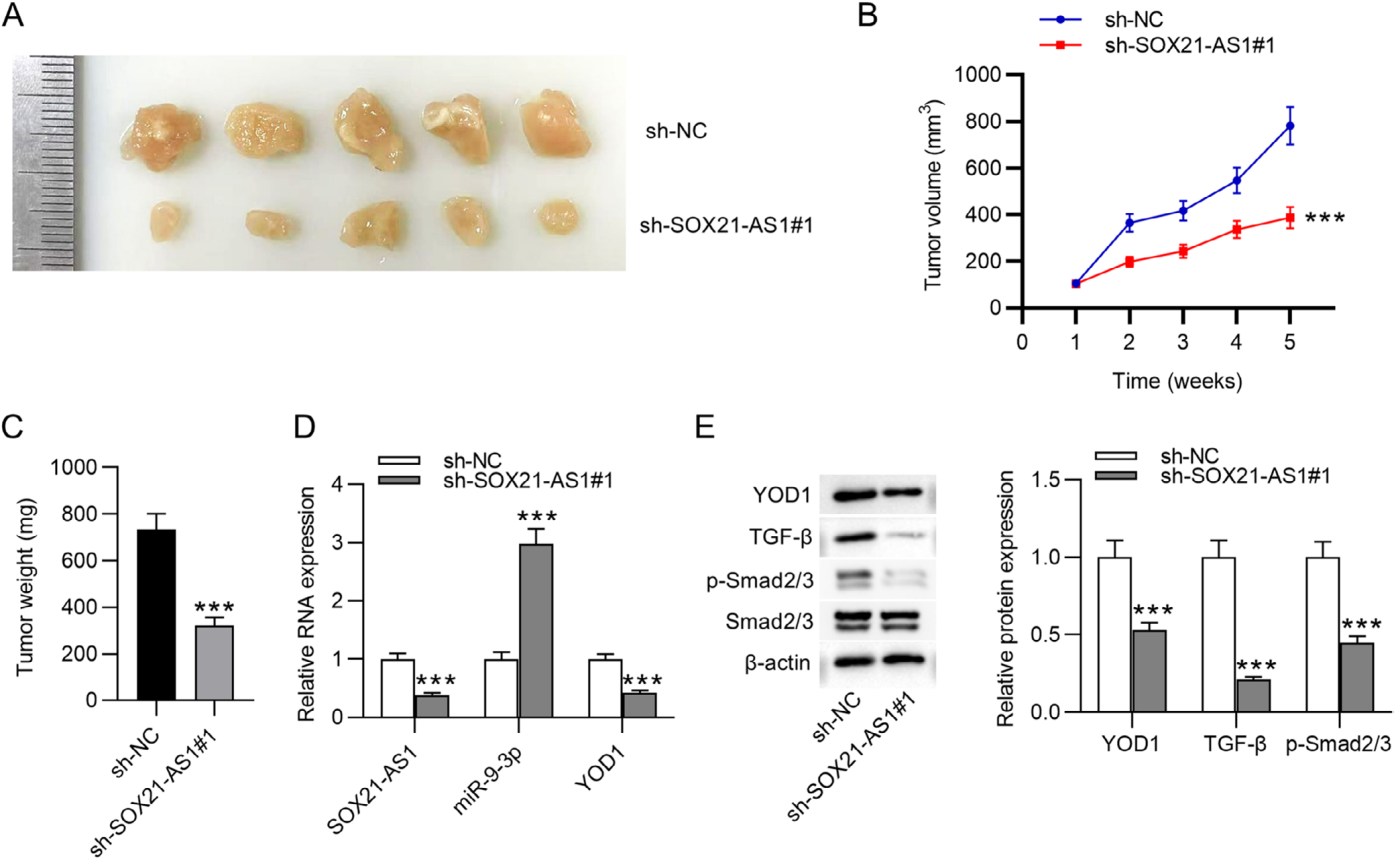
SOX21‐AS1 knockdown inhibited tumor growth in vivo. (A) Images of tumors harvested from mice in the sh‐NC and sh‐SOX21‐AS1#1 groups (*n* = 5 per group). (B) Tumor volume was measured weekly throughout the experimental period. (C) Final tumor weights recorded at the study endpoint. (D) qRT‐PCR analysis of SOX21‐AS1, miR‐9‐3p, and YOD1 expression levels in tumor tissues. (E) Western blot analysis of YOD1, TGF‐β, and phosphorylated Smad2/3 (p‐Smad2/3) protein levels in tumor samples. ***p < 0.001.

To further validate the oncogenic role of SOX21‐AS1 in PC, a xenograft mouse model was established by subcutaneously injecting PANC‐1 cells stably expressing either sh‐NC or sh‐SOX21‐AS1#1 into nude mice. Compared to the control group, mice in the sh‐SOX21‐AS1#1 group developed significantly smaller and slower‐growing tumors (Figure 7A–C), indicating that SOX21‐AS1 knockdown suppresses tumor growth in vivo. Further analysis using qRT‐PCR revealed that silencing SOX21‐AS1 resulted in upregulation of miR‐9‐3p and downregulation of YOD1 expression in tumor tissues (Figure 7D). Consistently, western blot analysis showed that SOX21‐AS1 knockdown led to reduced protein levels of YOD1, TGF‐β, and phosphorylated Smad2/3 (p‐Smad2/3) in the tumor samples (Figure 7E). These results support the conclusion that SOX21‐AS1 can promote PC tumor growth in vivo through modulation of the miR‐9‐3p/YOD1 axis and activation of the TGF‐β/Smad2/3 signaling pathway.

## 4 Discussion

PC is a highly lethal malignancy, with a median survival time of approximately 4 months and a 5‐year survival rate of only 13% [18]. While chemotherapy combined with surgical resection remains the primary therapeutic approach, only a limited number of patients qualify for surgical intervention [19]. Consequently, uncovering the molecular mechanisms underlying PC progression and identifying novel therapeutic targets are of critical importance. In this study, we demonstrated that the lncRNA SOX21‐AS1 facilitates the malignant phenotype of PC cells in vitro and promotes tumor growth in vivo. Mechanistically, SOX21‐AS1 functions as a ceRNA by sponging miR‐9‐3p, thereby upregulating YOD1 expression and activating the TGF‐β/Smad2/3 signaling pathway. These findings highlight SOX21‐AS1 as a potential oncogenic driver and therapeutic target in PC.

LncRNAs have been reported to be implicated in the regulation of diverse cellular processes, including proliferation, invasion, and apoptosis [20, 21]. Emerging studies have highlighted the oncogenic potential of SOX21‐AS1 across various malignancies. For instance, elevated SOX21‐AS1 expression has been detected in nephroblastoma patients, and its silencing was shown to inhibit the proliferative capacity of nephroblastoma cells [22]. Similarly, SOX21‐AS1 upregulation enhances stemness and invasive properties in breast cancer cells [23], and high expression levels are associated with poorer overall survival in cervical cancer patients [24]. Importantly, previous evidence has indicated the upregulation of SOX21‐AS1 in PC samples compared to normal controls [15]. In agreement with these findings, our study demonstrated that SOX21‐AS1 was significantly overexpressed in both PC cell lines and tumor tissues relative to non‐tumor controls. Functional loss‐of‐function assays revealed that silencing SOX21‐AS1 reduced cell viability and migration while promoting apoptosis in PC cells. Furthermore, in vivo experiments confirmed that SOX21‐AS1 knockdown effectively suppressed tumor growth in a xenograft mouse model. Collectively, these results reinforce the oncogenic role of SOX21‐AS1 in the progression of PC.

It is well‐established that lncRNAs can regulate downstream miRNAs to enhance the expression of their target mRNAs through a mechanism known as the ceRNA network [25]. Previous studies have shown that SOX21‐AS1 can influence disease progression via ceRNA‐mediated regulation [24, 26]. To elucidate the molecular mechanisms underlying the function of SOX21‐AS1 in PC, we employed a combination of bioinformatics analysis and experimental validation to identify its downstream effectors. Our findings revealed that SOX21‐AS1 directly binds to miR‐9‐3p, and that miR‐9‐3p targets YOD1 in PC cells. miR‐9‐3p has been previously reported to act as a tumor suppressor in various cancers, including gastric cancer and hepatocellular carcinoma [27, 28]. However, its expression profile and functional role in PC have not been fully characterized. In this study, we observed that miR‐9‐3p expression was significantly downregulated in both PC cells and tumor tissues compared to normal controls. Moreover, bioinformatics analysis indicated that higher miR‐9‐3p levels are associated with improved prognosis in PC patients. Additionally, a negative correlation between SOX21‐AS1 and miR‐9‐3p expression was detected in PC tissue samples, supporting the hypothesis that SOX21‐AS1 may contribute to PC progression by suppressing miR‐9‐3p expression.

YOD1, also known as OTUD2, is a highly conserved deubiquitinase belonging to the ovarian tumor (OTU) protease family. Previous studies have demonstrated diverse roles of YOD1 in disease progression. For instance, YOD1 suppresses neural precursor cell proliferation by deubiquitinating NEDD4 [29]. Han et al. reported that YOD1 facilitates the progression of triple‐negative breast cancer by stabilizing CDK1 [30]. Conversely, YOD1 has also been shown to inhibit the development of head and neck squamous cell carcinoma by preventing the ubiquitination and degradation of TRIM33 [31]. Importantly, prior research identified YOD1 as a promoter of PC cell proliferation and metastasis, as well as a potential prognostic biomarker [32]. Consistent with these findings, our study revealed that YOD1 was significantly upregulated in PC tissues compared to adjacent non‐tumorous tissues. Additionally, YOD1 expression exhibited a negative correlation with miR‐9‐3p and a positive correlation with SOX21‐AS1 levels in PC samples, supporting the hypothesis that SOX21‐AS1 acts as a ceRNA to upregulate YOD1 by sponging miR‐9‐3p. Notably, rescue experiments showed that overexpression of YOD1 effectively reversed the inhibitory effects of SOX21‐AS1 knockdown on PC cell proliferation, migration, and survival, thereby underscoring the critical role of the SOX21‐AS1/miR‐9‐3p/YOD1 regulatory axis in PC progression. The TGF‐β/Smad signaling pathway is a well‐established regulatory axis that plays a vital role in controlling cellular processes such as growth, migration, and differentiation [33]. This pathway is particularly significant in PC, where it contributes to both tumor progression and alterations in the tumor microenvironment [34]. Notably, previous studies have shown that YOD1 overexpression can enhance the expression of TGF‐β and promote Smad2/3 phosphorylation [35]. Conversely, silencing YOD1 has been reported to increase the ubiquitination of TGF‐β and p‐Smad2/3, thereby attenuating TGF‐β signaling [36]. A prior study also identified the TGF‐β pathway as a potential downstream effector through which YOD1 contributes to PC development [32]. In line with these findings, our study demonstrated that SOX21‐AS1 knockdown reduced TGF‐β expression and suppressed Smad2/3 phosphorylation in PC cells. These inhibitory effects were reversed upon YOD1 overexpression, indicating a regulatory relationship. Furthermore, YOD1 overexpression also restored the expression of fibrosis‐associated markers such as α‐SMA, fibronectin, and collagen I that were downregulated by SOX21‐AS1 silencing. These results suggest that SOX21‐AS1 promotes desmoplasia, a hallmark feature of PC, by upregulating YOD1 and activating the TGF‐β/Smad2/3 signaling pathway.

In conclusion, this study demonstrates that SOX21‐AS1 enhances the malignant behavior of PC cells in vitro and promotes tumor growth in vivo by modulating the miR‐9‐3p/YOD1 axis and activating the TGF‐β/Smad signaling pathway. These findings offer valuable insights into the molecular mechanisms driving PC progression and may contribute to the development of novel diagnostic markers and therapeutic strategies for PC.

## Ethics Statement

The study protocol was reviewed and approved by the Ethics Committee of Wuhan Third Hospital.

## Conflicts of Interest

The authors declare no conflicts of interest.

## Supporting information


**Figure S1.** qRT‐PCR analysis of SOX21‐AS1 and miR‐9‐3p expression in co‐transfected PC cells. (A) SOX21‐AS1 expression levels in cells transfected with SOX21‐AS1‐Wt or SOX21‐AS1‐Mut along with NC or miR‐9‐3p mimics. (B) miR‐9‐3p expression levels in the same groups. ***p < 0.001.

**Figure S2.** qRT‐PCR analysis of gene expression in transfected PC cells. Expression levels of SOX21‐AS1, miR‐9‐3p, YOD1, and TGF‐β were measured in cells transfected with sh‐NC, sh‐SOX21‐AS1#1, or co‐transfected with sh‐SOX21‐AS1#1 and pcDNA3.1/YOD1. ***p < 0.001.

## Data Availability

All data generated or analyzed during the current study are available from the corresponding author on reasonable request.
