# Supplementary figures and images for: LncRNA SOX21‐AS1 Promotes the Progression of Pancreatic Cancer by Sponging miR‐9‐3p and Upregulating YOD1
Source: Kaohsiung J Med Sci. 2025 Jun 17;41(10):e70054. doi: 10.1002/kjm2.70054 (PMC12520518; doi:10.1002/kjm2.70054)

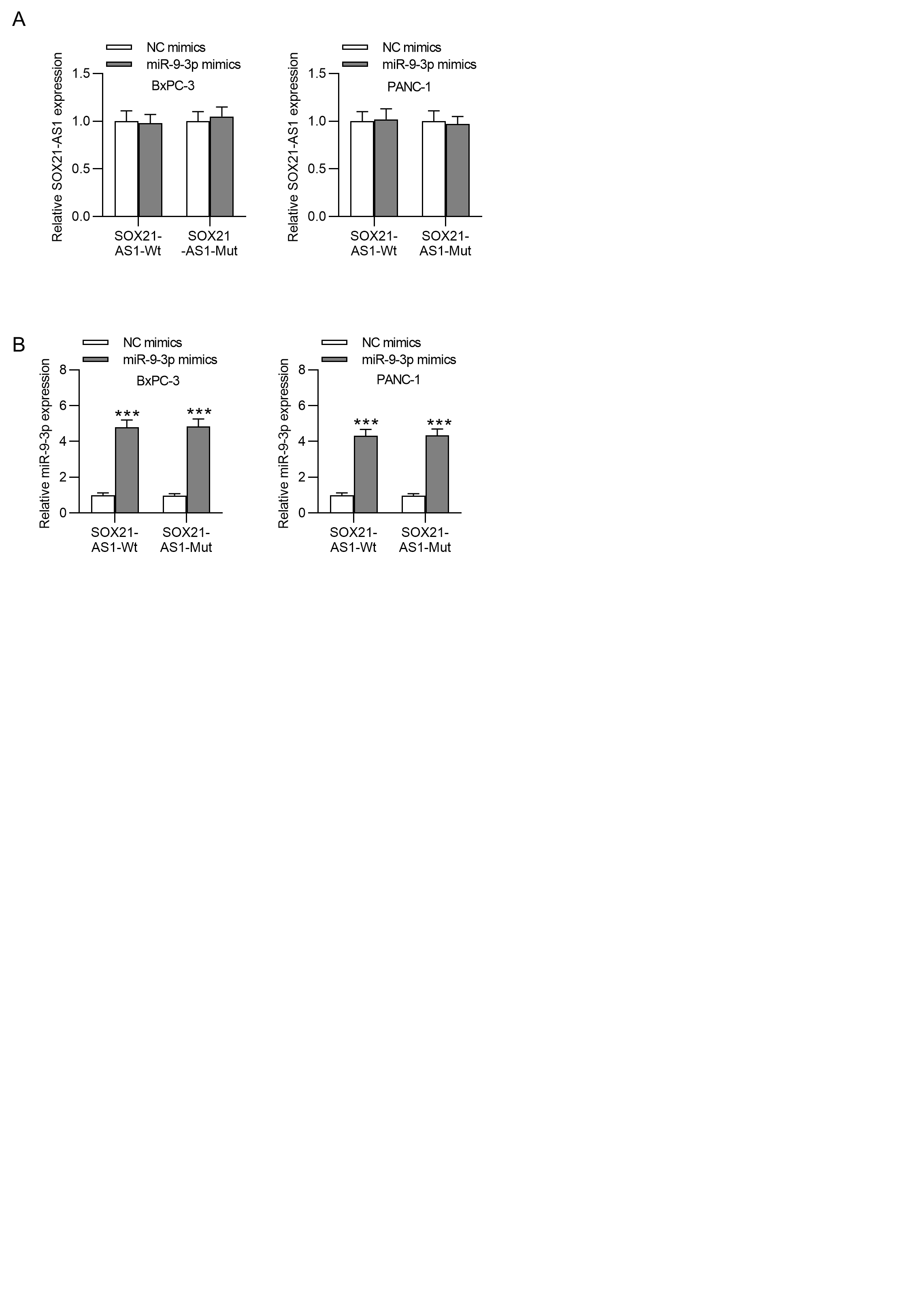

Supplement: Supplementary file 1 — Figure S1. qRT‐PCR analysis of SOX21‐AS1 and miR‐9‐3p expression in co‐transfected PC cells. (A) SOX21‐AS1 expression levels in cells transfected with SOX21‐AS1‐Wt or SOX21‐AS1‐Mut along with NC or miR‐9‐3p mimics. (B) miR‐9‐3p expression levels in the same groups. ***p < 0.001. [file KJM2-41-e70054-s002.tif]

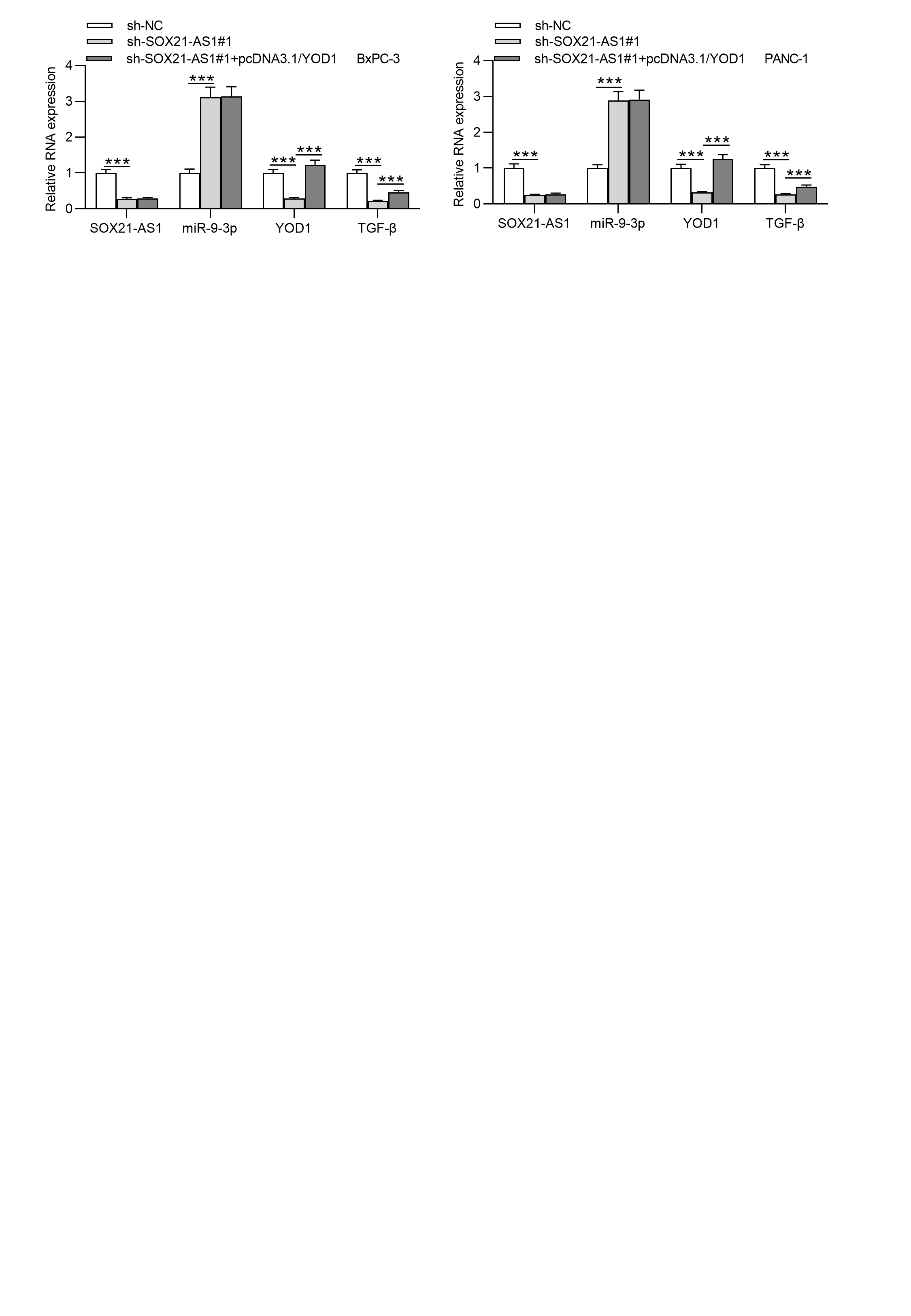

Supplement: Supplementary file 2 — Figure S2. qRT‐PCR analysis of gene expression in transfected PC cells. Expression levels of SOX21‐AS1, miR‐9‐3p, YOD1, and TGF‐β were measured in cells transfected with sh‐NC, sh‐SOX21‐AS1#1, or co‐transfected with sh‐SOX21‐AS1#1 and pcDNA3.1/YOD1. ***p < 0.001. [file KJM2-41-e70054-s001.tif]
